# Analysis of HA5 Hemagglutinins from Influenza A Viruses for Mass-Charge Patterns to Understand Antigenic Drift Related to Immune Escape

**DOI:** 10.3390/v18091025

**Published:** 2026-09-15

**Authors:** Wenhao Zhang, Xinran Wu, Rui Zhu, Xinyi Pan, Shancheng Yu, Jie Wang, Jun Steed Huang, Wandong Zhang

**Affiliations:** 1 School of Biomedical Engineering and Informatics, Nanjing Medical University, Nanjing 211166, China; wenhaozhang@stu.njmu.edu.cn; 2 School of Medical Imaging, Nanjing Medical University, Nanjing 211166, China; xinranwu@stu.njmu.edu.cn; 3 School of Rehabilitation Medicine, Nanjing Medical University, Nanjing 211166, China; 4 Faculty of Engineering, University of Ottawa, Ottawa, ON K1H 8M5, Canada; 5 School of Computer Science, Carleton University, Ottawa, ON K1S 5B6, Canada; jun.huang@carleton.ca; 6 Human Health Therapeutics Research Centre, National Research Council of Canada, Ottawa, ON K1A 0R6, Canada; 7 Faculty of Medicine, University of Ottawa, Ottawa, ON K1H 8M5, Canada

**Keywords:** influenza A viruses, wildlife, HA5 proteins, mass-charge patterns, predictive modelling algorithm, genetic and antigenic drift, immune escape

## Abstract

Influenza A viruses remain a persistent global health threat due to their ability to mutate and evade host immune defences, a process largely driven by changes in hemagglutinin (HA). HA is a glycoprotein present on the surface of influenza virions and plays a critical role in viral entry into host cells by binding to cellular receptors. In this study, 23 HA5 protein sequences from various influenza viral strains collected from wild birds, seagulls, ducks, geese, and chickens, as well as from humans in mainland China during the period from 2023 to 2024, were analyzed for mass-charge patterns by our predictive modelling algorithm to understand the nonlinear trend of viral evolution, particularly regarding their antigenic drift and immune escape potential. Two critical regions in HA5 proteins were identified and analyzed. The first region is a “mutable zone” (the negative gravity centre identified by mass-charge pattern analysis), which frequently accumulates multiple mutations. This leads to antigenic drift and instability, enabling the virus to evade pre-existing herd immunity. Analyzing and monitoring mass-charge changes may help predict the evolutionary trend of the viruses and the emergence of future variants in these regions where the viruses were collected, thereby informing the design and development of vaccines for the viruses analyzed and related strains. The second region is a “stable core” (positive gravity centre), a highly conserved region essential for viral function. Due to its conservation across strains, it may represent a promising target for developing universal vaccines and broad-spectrum antibodies capable of conferring protection against multiple variants simultaneously for the viruses analyzed and related strains. In summary, our analyses, with a predictive modelling algorithm for mass-charge patterns of HA5 protein sequences, provide a novel metric for viral evolution and evaluating the immune evasion potential, and offer a strategic framework for designing vaccines against the viruses analyzed and related strains.

## 1. Introduction

Influenza is an acute respiratory infectious disease caused by influenza viruses [1]. Based on their nucleoprotein and matrix protein, influenza viruses are classified into four types: A, B, C, and D [2]. According to the hemagglutinin (HA) and neuraminidase (NA) glycoproteins on the surface of influenza virions, the viruses can be further divided into numerous subtypes, such as H1N1 and H5N1 [3]. Over the past century, there have been four major influenza pandemics worldwide: the Spanish flu (in 1918), the Asian flu (in 1957), the Hong Kong flu (in 1968), and the H1N1 pandemic (in 2009) [4,5]. Among human populations, influenza A epidemics occur every 1~3 years, and influenza B outbreaks occur approximately every 3~5 years.

Infection with influenza virus damages respiratory mucosal epithelial cells and impairs their ability to adhere to and remove foreign substances, thereby significantly reducing respiratory function and resistance to infections. Consequently, influenza often leads to secondary infections, which pose a particular threat to vulnerable populations such as children, the elderly, and pregnant women [6]. Secondary pneumonia caused by influenza is one of the primary causes of influenza-related mortality. Statistics indicate that the 1918 Spanish flu resulted in approximately 40 to 50 million deaths, causing significant societal and economic disaster [7]. Nowadays, influenza mortality rates have declined substantially due to influenza vaccines and herd immunity. However, with the acceleration of globalization and increased population mobility, the risk of influenza transmission has further intensified. Strengthening global surveillance of influenza viruses, research on viral variations, vaccine development, and emergency preparedness are critical to combating influenza viruses.

Due to the tendency of HA and NA to undergo antigenic variation, among the three types of influenza viruses that infect humans, influenza A virus exhibits extremely high variability, followed by influenza B virus; while influenza C virus demonstrates highly stable antigenicity [8]. The extent of antigenic variation on the surface of influenza viruses resulting from antigenic drift and shift directly determines the scale of influenza outbreaks [9]. Minor variations, termed antigenic drift, generate new viral strains and can cause small to medium-sized epidemics. In contrast, major variations, known as antigenic shift, produce entirely new subtypes. In such cases, the population generally lacks immunity to these new subtypes, often leading to larger-scale epidemics or even worldwide pandemics [10]. Characterizing the mechanisms of antigenic drift and shift, as well as immune escape in influenza A viruses, is critical to understanding viral evolution and improving vaccine design and development [11,12,13]. Recent advances in understanding global epistasis and its role in shaping evolutionary dynamics emphasize that, although predicting mutational effects remains complex, emerging patterns offer promising avenues for evolutionary forecasting [14]. Predictive analyses of HA5 hemagglutinins in influenza A viruses, particularly those exploring epistatic drift in relation to immune escape, provide critical empirical support and underscore the broader applicability of these theoretical frameworks. Maurer et al. [15] employed deep mutational scanning to examine human antibody lineages targeting conserved hemagglutinin epitopes, demonstrating that while antibody affinity maturation restricts escape mutations in the initial imprinting strain, antigenic drift substantially broadens escape pathways in subsequent strains through complex epistatic interactions within HA. Their findings underscore the critical role of epistasis in influencing viral antigenic variability and immune evasion. The coordinated evolution of influenza surface glycoproteins, hemagglutinin and neuraminidase, has been widely investigated as a determinant of viral fitness and host immune system interactions. HA facilitates viral attachment to host cells, whereas NA promotes viral release, maintaining a functional balance between these proteins that is essential for efficient viral infection, replication, and virion release [16]. Mutational adjustments in NA can compensate for alterations in HA binding affinity, reflecting an epistatic co-adaptation process. Furthermore, HA stem-directed antibodies can inhibit NA enzymatic activity via steric hindrance, contributing to broad-spectrum immunity and highlighting the necessity of considering both antigens in vaccine development. Large-scale genomic sequencing efforts have significantly enhanced the understanding of influenza virus diversity and evolution. For example, whole-genome sequencing of more than 200 influenza A viral strains has revealed extensive genetic variation, including point mutations, deletions, and reassortment events [17]. The identification of recombination events leading to the 2003~2004 “Fujian-like” strain illustrates the importance of genomic surveillance in monitoring viral evolution and informing vaccine strain selection. Similarly, bioinformatic analyses of H5N1 viral HA sequences have identified mutation hotspots and regional clustering patterns, offering insights into viral transmission and adaptation, as well as immune escape [18]. Structural and functional studies further highlight the role of HA protein properties in viral infectivity and pathogenicity. For instance, mutations affecting HA amino acid stability alter viral replication efficiency and host immune responses in H1N1 models by modulating interferon pathways [19,20]. Crystallographic analyses of the H5N1 viral HA protein elucidated receptor specificity determinants and glycosylation sites involved in immune evasion, providing molecular-level insights into cross-species transmission potential [21]. Similar studies on early 2009 H1N1 isolates characterized antigenic sites and receptor-binding features critical for pandemic emergence [22]. Furthermore, studies demonstrate that the vestigial esterase (VE) domain of HA serves as a key antigenic site in H5N1 viruses. Antibodies targeting the VE domain exhibit cross-clade activity, block membrane fusion, and trigger Fc-dependent effects such as antibody-dependent cellular cytotoxicity (ADCC). Some of these antibodies confer superior protection compared to oseltamivir. VE residues influence HA acid stability and viral infectivity. These antibodies exhibit intermediate neutralization breadth, underscoring their potential for therapeutic and vaccine development [23]. Complementing these findings, risk evaluation platforms such as VarEPS-Influ integrate large-scale sequence data with multidimensional mutation impact assessments to predict functional consequences on viral proteins, including HA [24]. These tools facilitate early identification of potential immune escape variants and support public health responses.

**Figure 1:**
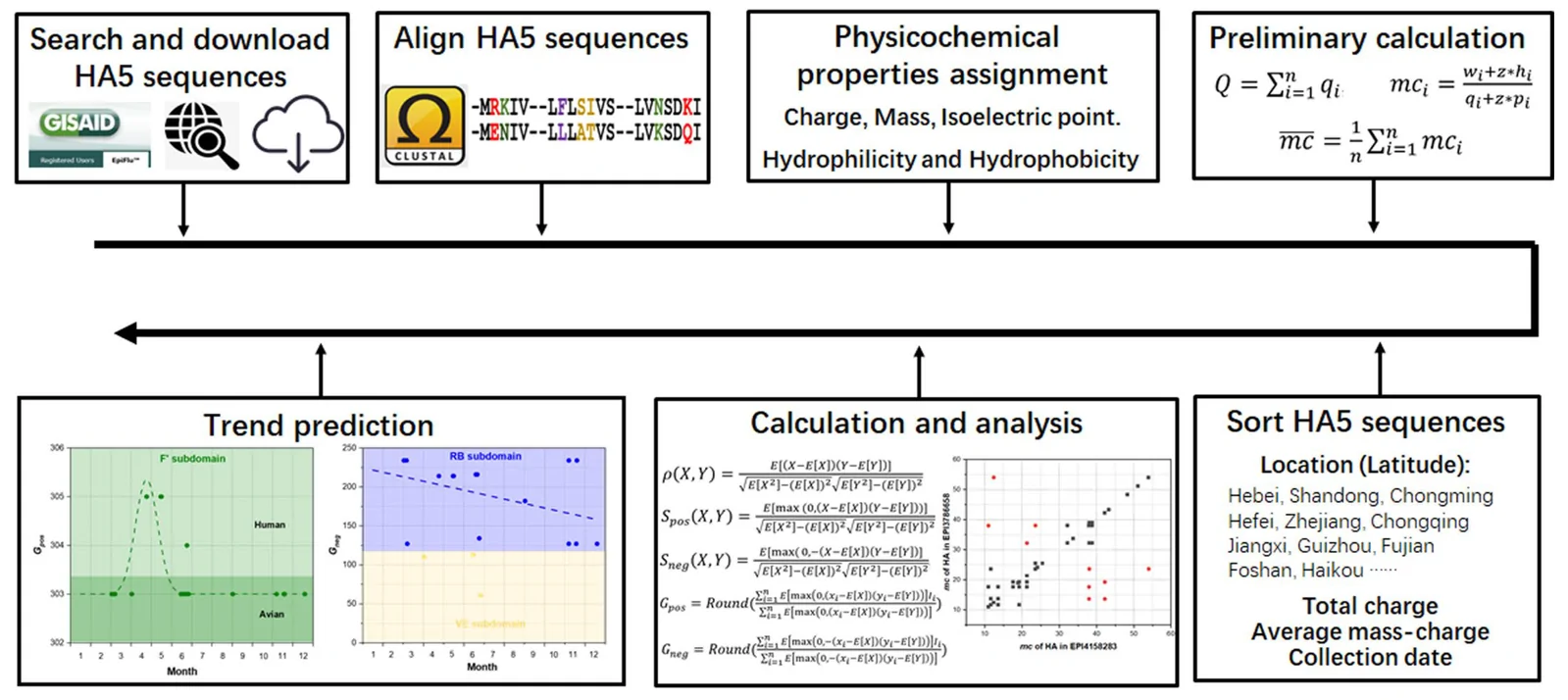
General study design workflow. The influenza A viral HA5 protein sequences were collected and aligned. The physiochemical properties of amino acids in the HA5 protein sequences were assigned as described in the Materials and Methods Section below. Preliminary calculations were conducted and the HA5 sequences were then assorted based on location (latitude) and collection date. Calculations and analysis were then performed, and viral evolution trend was predicted based on the analysis.

Typical analytical methods often encounter challenges such as manual inefficiency or excessive reliance on computational resources [25]. Huang et al. [26,27] developed a semi-covariance coefficient analytical tool by quantifying amino acid charges (+1, −1, or 0) and applying ReLU-based transformations to unfold epistatic relationships within spike proteins of SARS-CoV-2 and its variants. This approach successfully identified complex evolutionary trends related to viral infectivity, virulence, and immune escape, providing a robust tool for predicting viral evolutionary trajectories. In this study, our objectives are to examine the analogous charge-mass-specific relationship in binding protein sequences associated with viral immune escape. The general study design workflow is illustrated in Figure 1. We analyzed the evolution of influenza A viral HA5 sequences obtained from multiple sources in mainland China. Initially, viral RNA sequences undergo processing steps, including translation into protein sequences and alignment to ensure comparability. Each aligned sequence is then characterized by assigning key physicochemical properties to each amino acid residue, such as charge, mass, isoelectric point, hydrophilicity, and hydrophobicity. Subsequently, the mass-charge, Pearson correlation coefficient, semi-covariance coefficient, and gravity of HA sequences are computed and analyzed as described [26,27]. Finally, viral evolution trend prediction is performed by applying semi-covariance correlation to detect asymmetric or directional variations. This integrative approach may enable a comprehensive understanding of sequence biophysical properties and dynamics, facilitating the prediction of viral evolutionary trajectories.

## 2. Materials and Methods

The hemagglutinin (HA) protein sequences of influenza A viruses H5N1 and H5N6 used in this study were collected in geographically different regions of mainland China in different seasons of 2023 and 2024, including the spring, early summer, fall, and winter seasons. The data were obtained from the GISAID database, including the following Accession Numbers: EPI4158283, EPI3580932, EPI3567907, EPI4402483, EPI4404176, EPI4404168, EPI4404191, EPI3567810, EPI3567818, EPI3726722, EPI3520918, EPI3526751, EPI4402498, EPI4402490, EPI4399250, EPI4404183, EPI3567870, EPI2883780, EPI2882776, EPI3786658, EPI3786634, EPI3279875, and EPI3786561. Detailed information regarding the HA5 protein sequences is presented in Table S1, including 19 avian and 4 human HA5 protein sequences. Clustal Omega (https://www.ebi.ac.uk/jdispatcher/msa/clustalo?stype=protein accessed on 19 August 2026) (2024 version according to https://europepmc.org/article/MED/38597606 accessed on 19 August 2026) was employed to align the HA5 protein sequences from the selected influenza A viral strains. The aligned hemagglutinin protein sequences [labelled as X = ($x_{1}$,…, $x_{n}$)] were converted into charge sequences [labelled as q = ($q_{1}$,…, $q_{n}$), where +1 represents positively charged amino acids, −1 represents negatively charged amino acids, and 0 represents neutral amino acids]. Missing segments in the protein sequences were filled with the mean value. The total charge of the protein sequence is

(1) $$Q=\sum_{i=1}^{n}q_{i},$$

The mass-charge of the protein sequences [labelled as mc = ($mc_{1}$,…, $mc_{n}$)] can be calculated using the Langlands formula, which effectively integrates the influence of molecular mass, hydrophobicity, charge, and isoelectric point of the amino acid composition of the protein sequences on viral protein–host receptor interactions. Lanlands formula was not previously reported in biological studies, such as protein analysis. Because it can integrate the influence of the properties of amino acids and protein sequences as described above, the adoption of the formula in this study may be justified [28]. The formula can be expressed as follows:

(2) $${mc}_{i}=\frac{w_{i}+z*h_{i}}{q_{i}+z*p_{i}},$$

where $w_{i}$, $h_{i}$, $q_{i}$ and $p_{i}$ are the molecular mass, hydrophilic and hydrophobic index, charge, and isoelectric point of the amino acids in the hemagglutinin protein sequences; z is a scaling factor reflecting the Finsler distance between the viral protein and the host receptor. Typically, z values range from 1 to 10 (nm), with a default value of 1. Based on the literature indicating that the distance between the receptor-binding domain of the viral proteins and host receptors typically ranges from 1 to 10 nm [29,30,31], scaling factors (z) of 1, 3, 5, 7 and 10 were selected for mass-charge calculations. However, preliminary validation shows that although z values affect the absolute magnitude of mass-charge, they do not alter the drift patterns calculated by the semi-covariance method. Consequently, the example presented in the Results and Discussion Section was computed with z set to 1. The average mass-charge of the protein sequence is as follows:

(3) $$\bar{mc}=\frac{1}{n}\sum_{i=1}^{n}{mc}_{i},$$

The viral hemagglutinin protein sequences were subsequently sorted according to latitude, total charge, and average mass-charge of the protein sequences. In cases where these values remained identical, the sequences were further sorted by collection date. The linear correlation between the mass-charge of two viral sequences (X and Y) was quantified using the Pearson correlation coefficient (ρ), calculated as follows:

(4) $$\rho \left(X,Y\right)=\frac{E[\left(X-E\left[X\right]\right)\left(Y-E\left[Y\right]\right)]}{\sqrt{E\left[X^{2}\right]-{\left(E\left[X\right]\right)}^{2}}\sqrt{E\left[Y^{2}\right]-{\left(E\left[Y\right]\right)}^{2}}},$$

The nonlinear correlation between the mass-charge of two viral sequences was measured using the semi-covariance coefficient we previously proposed, which is defined as follows:

(5) $$S_{pos}\left(X,Y\right)=\frac{E[max(0,\;\left(X-E\left[X\right]\right)\left(Y-E\left[Y\right]\right))]}{\sqrt{E\left[X^{2}\right]-{\left(E\left[X\right]\right)}^{2}}\sqrt{E\left[Y^{2}\right]-{\left(E\left[Y\right]\right)}^{2}}},$$

(6) $$S_{neg}\left(X,Y\right)=\frac{E[\max(0,-\left(X-E\left[X\right]\right)\left(Y-E\left[Y\right]\right)]}{\sqrt{E\left[X^{2}\right]-{\left(E\left[X\right]\right)}^{2}}\sqrt{E\left[Y^{2}\right]-{\left(E\left[Y\right]\right)}^{2}}},$$

where $S_{pos}$ is the positive component of the semi-covariance coefficient and corresponds to the first and third quadrants, while $S_{neg}$ is the negative component of the semi-covariance coefficient and corresponds to the second and fourth quadrants. The max function is employed to introduce nonlinearity, enabling the capture of complex relationships within the data.

Gravity is used to identify the average position along the amino acid sequence where significant changes or mutations are concentrated. It helps pinpoint specific regions of the HA5 proteins that exhibit notable shifts in properties such as mass or charge, which may be important for understanding viral evolution and immune escape. To capture different types of changes, gravity is calculated separately for positive (convergent) and negative (divergent) trends in the data. These are represented by two values:

(7) $$G_{pos}=Round(\frac{\sum_{i=1}^{n}E\left[\max\left(0,\;\left(x_{i}-E\left[X\right]\right)\left(y_{i}-E\left[Y\right]\right)\right)\right]I_{i}}{\sum_{i=1}^{n}E\left[\max\left(0,\;\left(x_{i}-E\left[X\right]\right)\left(y_{i}-E\left[Y\right]\right)\right)\right]}),$$

(8) $$G_{neg}=Round(\frac{\sum_{i=1}^{n}E\left[\max\left(0,-\left(x_{i}-E\left[X\right]\right)\left(y_{i}-E\left[Y\right]\right)\right)\right]I_{i}}{\sum_{i=1}^{n}E\left[\max\left(0,-\left(x_{i}-E\left[X\right]\right)\left(y_{i}-E\left[Y\right]\right)\right)\right]}),$$

where $G_{pos}$ is the convergent component of gravity, and $G_{neg}$ is the divergent component of gravity; $I_{i}$ represents the position in the sequence, and Round denotes the rounding operation. By distinguishing between positive and negative contributions, these gravity measures offer a clear spatial representation of regions along the HA5 protein sequence where significant evolutionary changes cluster, facilitating understanding of viral adaptative mechanisms.

## 3. Results and Discussion

**Figure 2:**
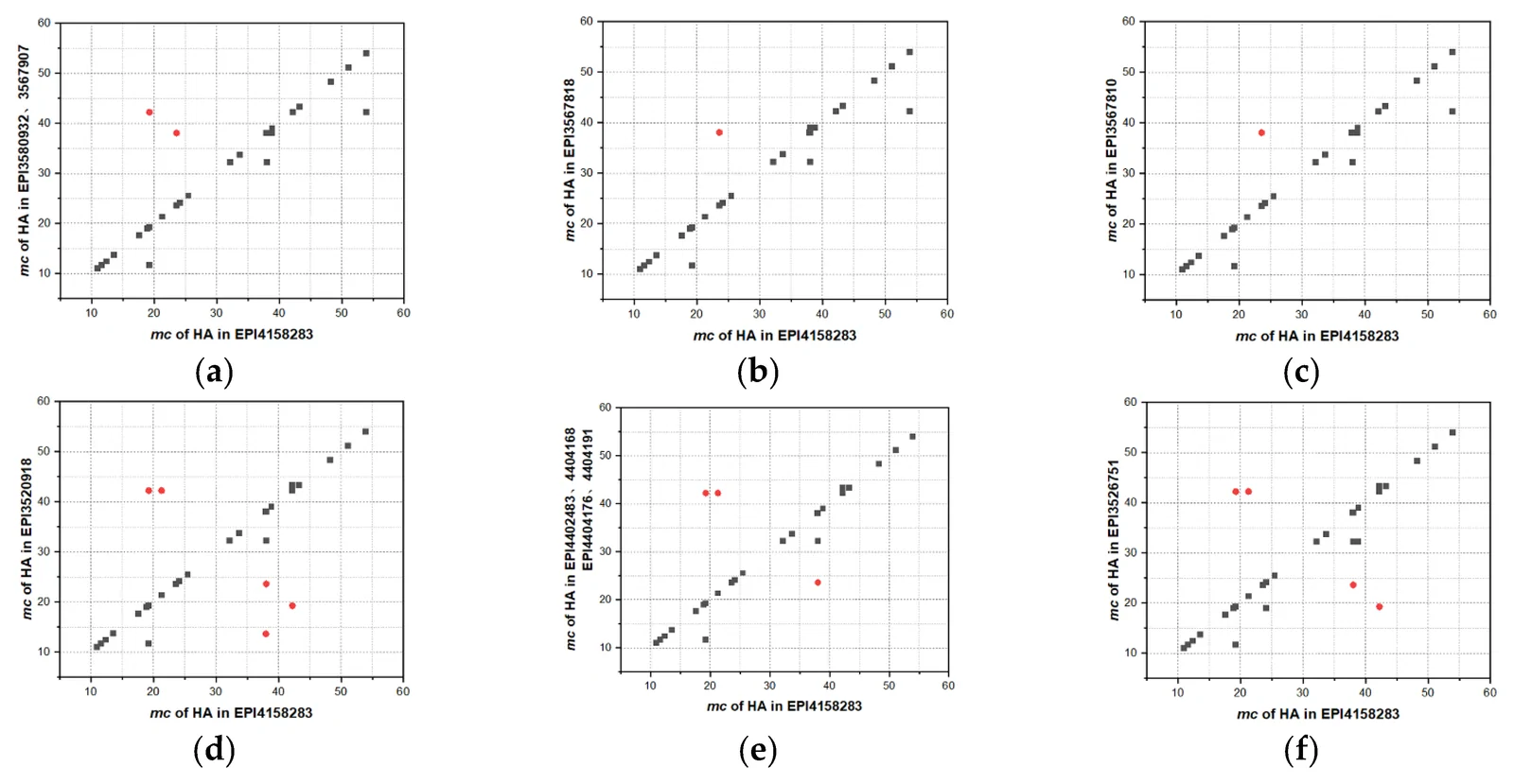

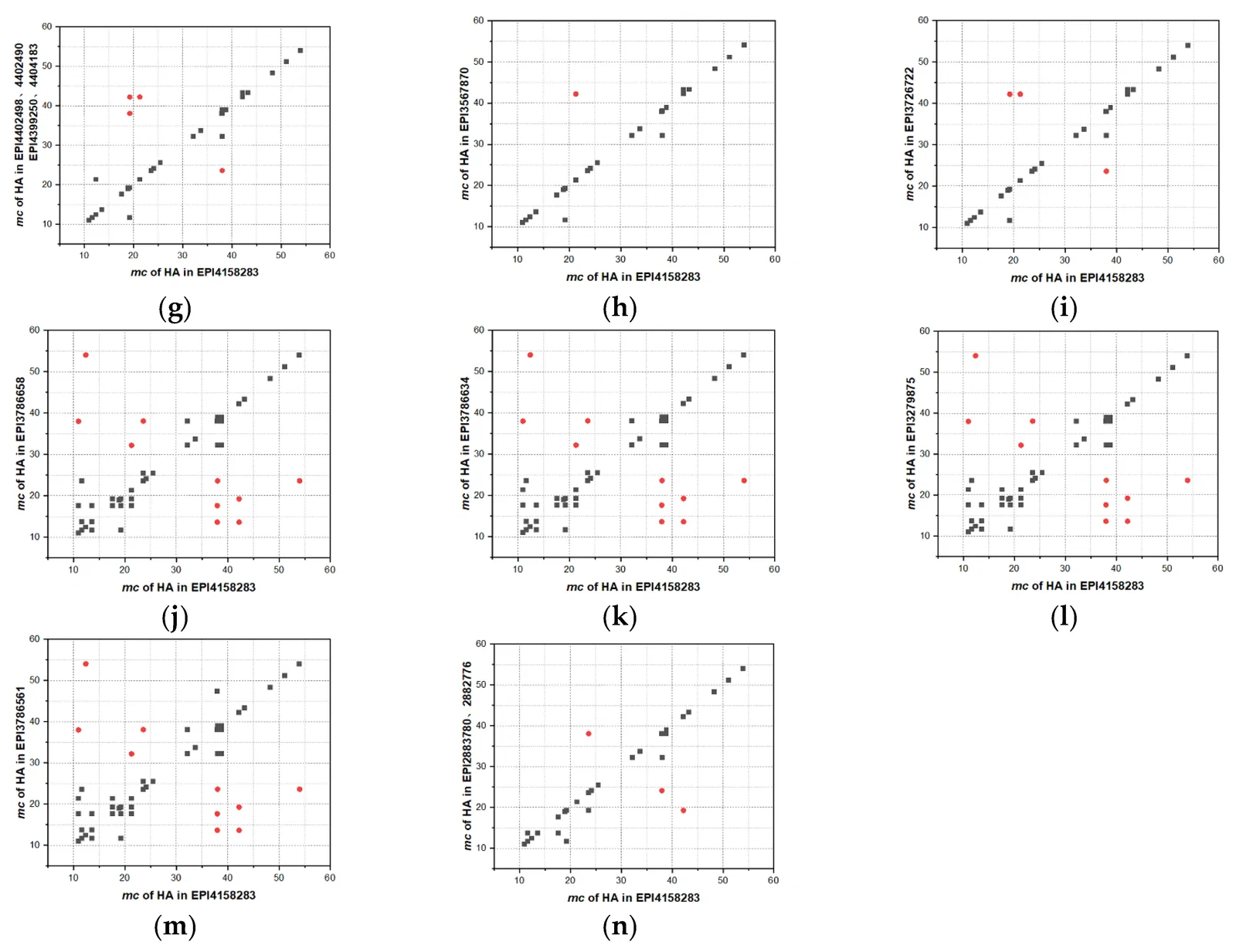
Visualization of mass-charge drift of the HA5 protein sequences (■: positive drift, ●: negative drift). The mass-charge drift of ((**a**) EPI3580932, EPI3567907), ((**b**) EPI367818), ((**c**) EPI3567810), ((**d**) EPI3520918), ((**e**) EPI4402483, EPI4404168, EPI4404176, EPI4404191), ((**f**) EPI3526751), ((**g**) EPI4402498, EPI4402490, EPI4399250, EPI4404183), ((**h**) EPI3567870), ((**i**) EPI3726722), ((**j**) EPI3786658), ((**k**) EPI3786634), ((**l**) EPI3279875), ((**m**) EPI3786561), ((**n**) EPI2883780, EPI2882776) strain compared with EPI4158283 strain.

First, the total charge, mass-charge and average mass-charge for each HA5 protein sequence were calculated using formulae 1, 2, and 3. The sequences were then sorted based on their sampling latitude, total charge, average mass-charge, and collection date. According to the sorting results presented in Table S2, EPI4158283 (A/Seagull/Hebei/qhd6/2024_HA) was selected as the initial HA5 protein sequence for the computation. By comparing it with the initial HA5 protein sequence, the nominal mass-charge change in the protein sequence under study was obtained (shown in Figure 2), thereby enabling investigation of the drift patterns associated with immune escape. HA5 proteins with identical amino acid sequences were plotted in the same subplot, such as Figure 2a for EPI3580932 and EPI3567907. The number of negative drifts exhibits some correlation with factors such as host species and latitude difference. For example, the range of variation is 1~4 for avian hosts and 5~10 for human hosts, with an increasing trend observed as the latitude difference expands (listed in Table S3). These visualizations identify distinct regions within the HA5 protein sequence where mass-charge values display increasing or decreasing trends, indicating localized mutational changes that may influence viral binding properties and antigenicity.

Table S3 also presents the semi-covariance coefficients and gravity drift metrics that quantify the nonlinear correlations between HA5 sequences from the EPI4158283 strain (Qinhuangdao designated as the primary site) and other strains identified from different locations. During avian transmission, the HA5 proteins demonstrate significant sequence conservation, as indicated by high positive semi-covariance coefficients ($S_{pos}$ > 0.994) and low negative semi-covariance coefficients ($S_{neg}$ < 0.004). The Pearson correlation coefficients (ρ) exceeding 0.99 indicate strong linear relationships between sequences. In contrast, cross-species transmission to humans reveals distinct sequence divergence, characterized by positive semi-covariance coefficients below 0.994 and negative semi-covariance coefficients above 0.005. Notably, with increasing latitudinal distance, the positive semi-covariance coefficient decreases to 0.977543, while the negative semi-covariance coefficient increases to 0.014605, accompanied by a reduction in the Pearson correlation coefficient to 0.962938, indicating substantially diminished linear correlation. The gravity centres for positive drifts ($G_{pos}$) cluster tightly at amino acid residues 303~305, suggesting this region with neighbouring area represents a highly conserved functional domain in the HA5 protein. Conversely, the gravity centres for negative drifts ($G_{neg}$) are broadly distributed across residues 61~234, forming prominent mutational hotspots that may represent key sites mediating viral antigenic drift for immune escape. The gravity centres of negative drifts identified in our analysis overlap with the mutation hotspots identified by genetic and MS studies in human influenza viral HA sequences [13,32,33], which indirectly validates our analyses.

**Figure 3:**
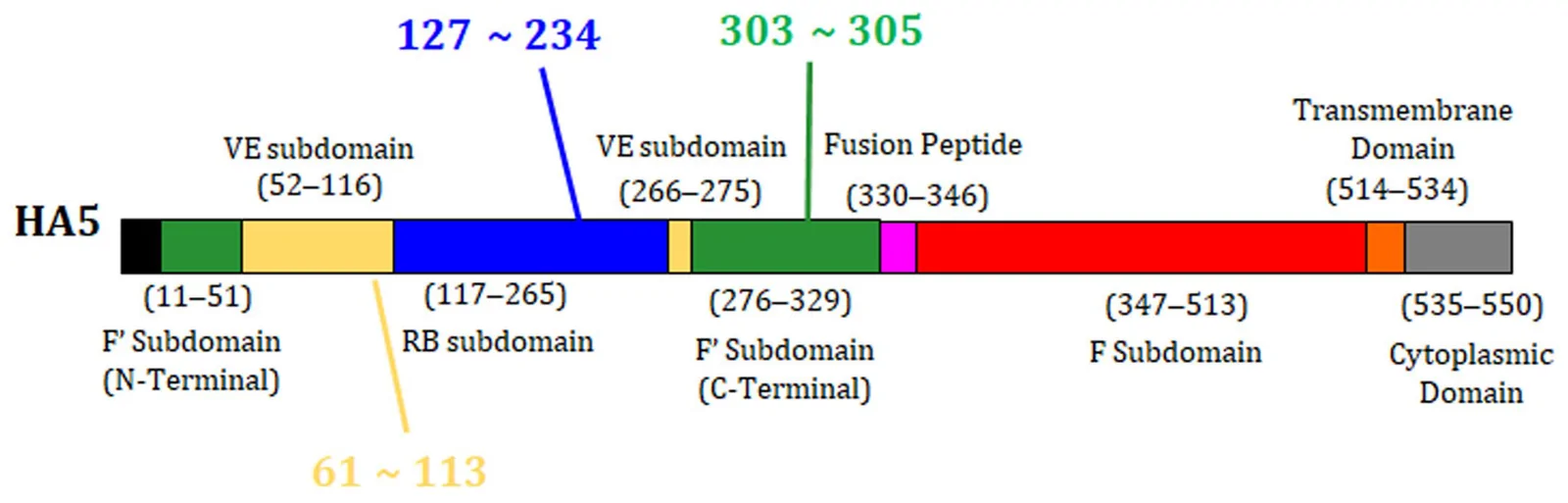
Corresponding motif drifts of HA5 protein sequences adopted from Zheng et al. 2018 [23].

To contextualize our predictive results, we reference the domain structure of the hemagglutinin protein as mapped in Figure 1A of Zheng et al. [23], which highlights the major functional subdomains of the HA5 subtype. As shown in Figure 3, all HA5 protein sequences analyzed in this study exhibit their positive drift gravity centres localized to the F′ subdomain (marked in green), while the negative drift gravity centres are distributed across the vestigial esterase (VE, marked in yellow) and receptor-binding (RB, marked in blue) subdomains. The F′ subdomain within the stalk region has gained attention as a structurally conserved and functionally significant region, which may be a critical target for next-generation influenza vaccines and therapeutic antibodies. The VE subdomain, located in the globular head region and evolutionarily derived from an ancient esterase, may still play a significant structural role in mediating membrane fusion and viral entry into host cells despite the loss of its original enzymatic activity. In contrast, the RB subdomain, located in the globular head, remains a hotspot for adaptive mutations that influence host specificity and facilitate zoonotic transmission. Grounded in this structural framework, our analysis of drift quantified through nominal mass-charge variations across the HA5 sequences reveals directional shifts in amino acid properties that reflect nonlinear evolutionary dynamics. Mapping these quantified drift sites onto structural subdomains provides mechanistic insight into the selective forces shaping antigenic evolution.

**Figure 4:**
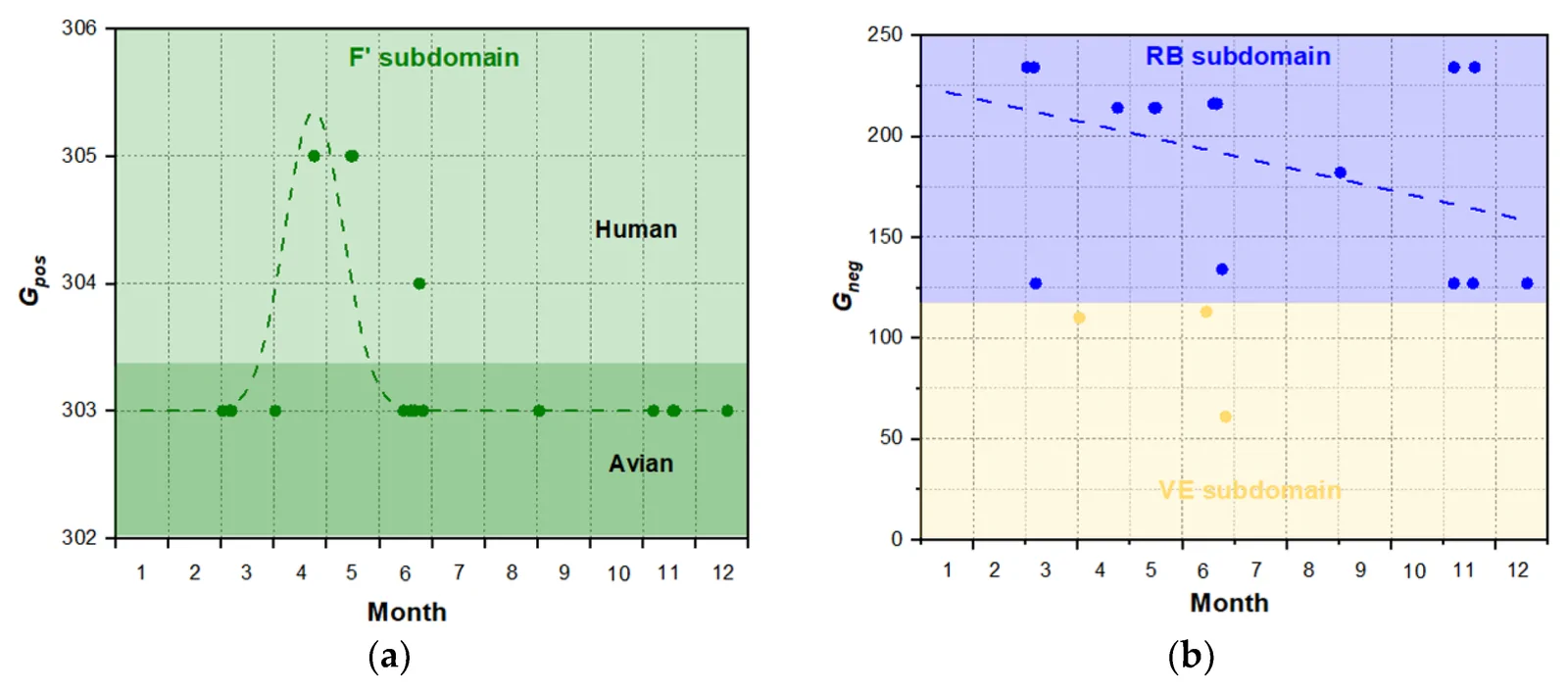
Trend prediction based on the months: ● Green colour represents F’ subdomain; ● Blue colour represents RB subdomain; and ● Light orange colour represents VE subdomain. Panel (**a**) shows the positive trends of gravity, and panel (**b**) shows the negative trends of gravity.

Figure 4 demonstrates the potential application of the semi-covariance method for predicting the monthly evolutionary trajectory of HA5 proteins for these collected viruses. The gravity centres of positive drifts for all avian strains consistently reside at residue 303, reflecting high evolutionary conservation. Notably, during the April–June period, heightened cross-species transmission activity to humans is observed, with the gravity centres of positive drifts shifting to residues 304~305. The gravity centres of negative drifts display extensive distribution across residues 61~234, with most located in the RB subdomain and showing a negative correlation with month (correlation coefficient: −0.43). It is particularly noteworthy that during the April–June period, the negative drifts exhibited a unique pattern involving the relatively conserved VE subdomain. This suggests that the evolutionary dynamics of typically conserved regions may be subjected to seasonal regulatory mechanisms. Collectively, these findings support the utility of semi-covariance coefficient analysis combined with mass-charge drift visualization as a robust framework for characterizing viral protein evolution related to immune escape mechanisms. This approach offers valuable predictive insights that may inform vaccine design strategies targeting emerging influenza variants. It could enable the identification of multiple influenza viruses with established vaccines that resemble the virus to be prevented, based on the positive and negative drift of gravity centres calculated by the semi-covariance method, rather than the amino acid residues of the HA5 protein sequences. The combination of these established vaccines could potentially address the persistent lag in clinical trials for new vaccines relative to viral epidemics. As evidenced by the entire computational process, our approach effectively circumvents issues commonly encountered with typical analytical methods, such as the inefficiency of manual phylogenetic inference [34] and the heavy reliance on substantial data and computational resources required for deep mutational scanning [15,35].

Limitations of the study and future directions: There are limitations in our study due to limited scope, resources, and manpower for this work: (1) Sample/data collection: Twenty-three influenza viral HA5 protein sequences were collected from the GISAID database and used in our study. Other viral subtypes were not included in the study, such as HA1, HA3, HA7, etc. The regions and latitude of these viral collections are only limited to mainland China, and do not apply globally. Thus, the results of the study may not be generalized, and bias may not be eliminated due to these limitations. (2) The study relies on sequence-based features or one-dimensional HA5 protein sequence information, and 3D structural features of HA5 proteins were not considered. In addition, the semi-covariance coefficient analysis in this study is not benchmarked against other classic nonlinear algorithms. (3) Viral neuraminidase (NA) also plays an important role in viral pathogenesis and evolution. NA co-evolution in antigenic drift/shift was not considered in this study. (4) This study contains correlational analyses based on collected influenza viral HA5 protein sequences. However, in vitro functional assays and validation were not conducted. In our next-step studies, more geographically collected viral strains and subtypes will be included to eliminate potential bias. Viral neuraminidases will be included in the analyses for co-evolution with HA proteins for antigenic drift and shift in immune escape. Three-dimensional structural features of HA and NA proteins will be considered in the analysis. AI-based methods and tools will be used to improve modelling and to minimize or eliminate potential bias. Forna et al. reported an approach of alignment-free prediction of cross-reactivity in influenza A (H3N2) to anticipate antigenic drift [36]. This method may also be used to validate our findings in future studies. The application of semi-covariance coefficient analysis will be benchmarked against other classic nonlinear algorithms. More importantly, in vitro functional and mutational assays will be conducted to validate the findings from these mass-charge model analyses [37].

## 4. Conclusions

In this study, we examined the mass-charge patterns of HA5 protein sequences from avian and human influenza A viruses collected in mainland China during the period from 2023 to 2024. Our analyses indicate that both average value and gravity centre position may predict the mutation characteristics of HA5 protein sequences that are associated with viral immune escape in the viruses analyzed and related strains. These metrics may assess the immune escape capability of currently circulating variants and predict the evolutionary trends of these viral strains as well as future emerging variants in mainland China. Specifically, the negative gravity centre spans amino acid positions 61 to 234, a region where mutations occur frequently; whereas the positive gravity centre is located in the narrow region from amino acid positions 303 to 305. The positive centre and the neighbouring area are highly conserved during evolution. Frequent mutations in the negative gravity centre suggest antigenic instability or antigenic drift in this region. Mutations leading to antigenic drift in this region enable the virus or its variants to evade pre-existing herd immunity, acquiring new antigenic properties that may lead to endemic spread in the regions where the viruses were collected. Analysis of this region may predict future changes or antigenic drift in the viral HA5 proteins, influencing viral infectivity and immune escape capability in these viruses analyzed and related strains. In contrast, the positive gravity centre, i.e., the highly conserved region of HA5 proteins, is critical to protein function and exhibits stable antigenic properties. Mutations in this region may be deleterious to the virus. Targeting this region for vaccine and antibody development could provide cross-protective immunity against relevant viral strains [38]. Thus, this semi-covariance coefficient analysis may be useful not only for predicting immune escape potential in influenza A viral HA5 proteins but also for providing information for vaccine and antibody development against these viruses analyzed and related strains [39]. This study has several limitations as described above. Future studies will address these limitations.

## Data Availability

The calculation formulas are provided in this paper, and data will be available on request.

## Supplementary Materials

The following supporting information can be downloaded at: https://www.mdpi.com/article/10.3390/v18091025/s1, Table S1: The information of hemagglutinin protein sequences; Table S2: The sorting of HA5 protein sequences; Table S3: The mass-charge drift, semi-covariance coefficient and gravity drift of the HA5 protein sequences.
