# Supplementary material for: Analysis of HA5 Hemagglutinins from Influenza A Viruses for Mass-Charge Patterns to Understand Antigenic Drift Related to Immune Escape
Source: Viruses. 2026 Sep 15;18(9):1025. doi: 10.3390/v18091025 (PMC13612040; doi:10.3390/v18091025)
Supplement: Supplementary file 1 [file viruses-18-01025-s001.zip › viruses-4471460-supplementary.pdf]

Table S1. The information of hemagglutinin protein sequences.

Accession#	Identifier	Virus Isolate ID	Type	Collection date	Host	Location	Latitude
EPI4158283	A/Seagull/Hebei/qhd6/2024_HA	EPI_ISL_19795829	H5N1	2024-12-01	Avian (Seagull)	Hebei (Qinhuangdao)	39.9658
EPI3580932	20240923YZ06HA	EPI_ISL_19450594	H5N1	2024-06-25	Avian (Live poultry market)	Shandong (Yanzhou)	35.5742
EPI3567907	20240923YZ006HA	EPI_ISL_19433138	H5N1	2024-06-25	Avian (Live poultry market)	Shandong (Yanzhou)	35.5742
EPI4402483	A/Anas crecca/Chongming/H5N1/2024_HA	EPI_ISL_19890473	H5N1	2024-11-06	Duck (Anas crecca)	Chongming Municipality	31.5587
EPI4404176	A/Anas poecilorhyncha/Chongming/H5N1_1/2024_HA	EPI_ISL_19890772	H5N1	2024-11-17	Duck (Anas poecilorhyncha)	Chongqing Municipality	29.5581
EPI4404168	A/Anas platyrhynchos/Chongming/H5N1_1/2024_HA	EPI_ISL_19890771	H5N1	2024-11-17	Duck (Anas platyrhynchos)	Chongqing Municipality	29.5581
EPI4404191	A/Anas platyrhynchos/Haikou/H5N1/2024_HA	EPI_ISL_19890774	H5N1	2024-12-18	Duck (Anas platyrhynchos)	Hainan (Haikou)	19.9385
EPI3567810	20240923_JinX06HA	EPI_ISL_19432676	H5N2	2024-06-20	Chicken	Shandong (Jinxiang)	35.0747
EPI3567818	20240923_WS104HA	EPI_ISL_19432677	H5N1	2024-06-18	Chicken	Shandong (Weishan)	35.0765
EPI3726722	2.4	EPI_ISL_19616579	H5N1	2024-03-06	Duck	Guizhou	26.6471
EPI3520918	A/HeFei04171/2024	EPI_ISL_19356984	H5N6	2024-06-23	Human	Anhui (Hefei)	31.7958
EPI3526751	A/wild bird/Zhejiang/NBXS-1/2024	EPI_ISL_19371693	H5N6	2024-06-14	Wild bird	Zhejiang	30.2632
EPI4402498	A/Mareca penelope/Jiujiang/H5N1/2024_HA	EPI_ISL_19890475	H5N1	2024-03-01	Duck (Mareca Penelope)	Jiangxi (Jiujiang)	29.7198
EPI4402490	A/Anas platyrhynchos/Jiujiang/H5N1/2024_HA	EPI_ISL_19890474	H5N1	2024-11-06	Duck (Anas platyrhynchos)	Jiangxi (Jiujiang)	29.7198
EPI4399250	A/Anser fabalis/Jiujiang/H5N1/2024_HA	EPI_ISL_19886139	H5N1	2024-03-05	Goose (Anser fabalis)	Chongqing Municipality	29.5581
EPI4404183	A/Spatula querquedula/Chongming/H5N1_1/2024_HA	EPI_ISL_19890773	H5N1	2024-11-18	Duck (Spatula querquedula)	Chongqing Municipality	29.5581
EPI3567870	A/duck/Jiangxi/631/2024_HA	EPI_ISL_19432687	H5N1	2024-04-01	Duck	Jiangxi	29.2488
EPI2883780	A/goose/China/FOSU-ZQ25/2023_HA	EPI_ISL_18718559	H5N6	2023-09-01	Goose	Foshan	23.0214
EPI2882776	A/goose/China/FOSU-ZQ28/2023_HA	EPI_ISL_18718124	H5N6	2023-09-01	Goose	Foshan	23.0214
EPI3786658	A/Quanzhou/2Env5/2024	EPI_ISL_19661726	H5N6	2024-05-15	Surface swab	Fujian	24.8739
EPI3786634	A/Quanzhou/2/2024	EPI_ISL_19661712	H5N6	2024-05-14	Human	Fujian	24.8739
EPI3279875	A/Fujian/1/2024(H5N6)	EPI_ISL_19132453	H5N6	2024-04-23	Human	Fujian (Quanzhou)	24.8739
EPI3786561	A /Quanzhou/1/2024	EPI_ISL_19661711	H5N6	2024-04-23	Human	Fujian	24.8739

Table S2. The sorting of HA5 protein sequences.

Accession#	Latitude	Total charge	Average mass-charge	Collection date
EPI4158283	39.9658	11.23280423	28.57004865	2024-12-01
EPI3580932	35.5742	11.23280423	28.58944001	2024-06-25
EPI3567907	35.5742	11.23280423	28.58944001	2024-06-25
EPI3567818	35.0765	12.25396825	28.55226463	2024-06-18
EPI3567810	35.0747	12.25396825	28.54936793	2024-06-20
EPI3520918	31.7958	13.27513228	28.51577052	2024-06-23
EPI4402483	31.5587	11.23280423	28.59919396	2024-11-06
EPI3526751	30.2632	13.27513228	28.53817318	2024-06-14
EPI4402498	29.7198	10.21164021	28.65008496	2024-03-01
EPI4402490	29.7198	10.21164021	28.65008496	2024-11-06
EPI4399250	29.5581	10.21164021	28.65008496	2024-03-05
EPI4404183	29.5581	10.21164021	28.65008496	2024-11-18
EPI4404176	29.5581	11.23280423	28.59919396	2024-11-17
EPI4404168	29.5581	11.23280423	28.59919396	2024-11-17
EPI3567870	29.2488	13.27513228	28.57077608	2024-04-01
EPI3726722	26.6471	12.25396825	28.58534704	2024-03-06
EPI3786658	24.8739	15.34452297	28.4986683	2024-05-15
EPI3786634	24.8739	15.34452297	28.51693273	2024-05-14
EPI3279875	24.8739	16.36749117	28.5158647	2024-04-23
EPI3786561	24.8739	16.36749117	28.53231591	2024-04-23
EPI2883780	23.0214	14.2962963	28.4921631	2023-09-01
EPI2882776	23.0214	14.2962963	28.4921631	2023-09-01
EPI4404191	20.0273	11.23280423	28.59919396	2024-12-18

Table S3. The mass-charge drift, semi-covariance coefficient and gravity drift of the HA5 protein sequences.

Initial / Terminal HA5 for computation	Initial / Terminal Host	Initial / Terminal Type	Sampling month	Latitude difference	Number of negative drifts	S_{pos}	G_{pos}	S_{neg}	G_{neg}	ρ
EPI4158283 / EPI3580932	Avian (Seagull) / Avian (Live poultry market)	H5N1 / H5N1	06-25	4.3916	2	0.996983	303	0.001695	61	0.995288
EPI4158283 / EPI3567907	Avian (Seagull) / Avian (Live poultry market)	H5N1 / H5N1	06-25	4.3916	2	0.996983	303	0.001695	61	0.995288
EPI4158283 / EPI3567818	Avian (Seagull) / Chicken	H5N1 / H5N1	06-18	4.8893	1	0.998315	303	0.00046	216	0.997855
EPI4158283 / EPI3567810	Avian (Seagull) / Chicken	H5N1 / H5N2	06-20	4.8911	1	0.998316	303	0.000461	216	0.997855
EPI4158283 / EPI3520918	Avian (Seagull) / Human	H5N1 / H5N6	06-23	8.17	5	0.993666	304	0.005247	134	0.988419
EPI4158283 / EPI4402483	Avian (Seagull) / Duck (Anas crecca)	H5N1 / H5N1	11-06	8.4071	3	0.996504	303	0.002655	127	0.993849
EPI4158283 / EPI3526751	Avian (Seagull) / Wild bird	H5N1 / H5N6	06-14	9.7026	4	0.994829	303	0.003893	113	0.990936
EPI4158283 / EPI4402498	Avian (Seagull) / Duck (Mareca Penelope)	H5N1 / H5N1	03-01	10.246	4	0.995257	303	0.003506	234	0.991751
EPI4158283 / EPI4402490	Avian (Seagull) / Duck (Anas platyrhynchos)	H5N1 / H5N1	11-06	10.246	4	0.995257	303	0.003506	234	0.991751
EPI4158283 / EPI4399250	Avian (Seagull) / Goose (Anser fabalis)	H5N1 / H5N1	03-05	10.4077	4	0.995257	303	0.003506	234	0.991751
EPI4158283 / EPI4404183	Avian (Seagull) / Duck (Spatula querquedula)	H5N1 / H5N1	11-18	10.4077	4	0.995257	303	0.003506	234	0.991751
EPI4158283 / EPI4404176	Avian (Seagull) / Duck (Anas poecilorhyncha)	H5N1 / H5N1	11-17	10.4077	3	0.996504	303	0.002655	127	0.993849
EPI4158283 / EPI4404168	Avian (Seagull) / Duck (Anas platyrhynchos)	H5N1 / H5N1	11-17	10.4077	3	0.996504	303	0.002655	127	0.993849
EPI4158283 / EPI3567870	Avian (Seagull) / Duck	H5N1 / H5N1	04-01	10.717	1	0.998107	303	0.000963	110	0.997144
EPI4158283 / EPI3726722	Avian (Seagull) / Duck	H5N1 / H5N1	03-06	13.3187	3	0.996227	303	0.002654	127	0.993574
EPI4158283 / EPI3786658	Avian (Seagull) / Surface swab	H5N1 / H5N6	05-15	15.0919	10	0.978472	305	0.014599	214	0.963874
EPI4158283 / EPI3786634	Avian (Seagull) / Human	H5N1 / H5N6	05-14	15.0919	10	0.977928	305	0.014622	214	0.963306
EPI4158283 / EPI3279875	Avian (Seagull) / Human	H5N1 / H5N6	04-23	15.0919	10	0.977944	305	0.014619	214	0.963325
EPI4158283 / EPI3786561	Avian (Seagull) / Human	H5N1 / H5N6	04-23	15.0919	10	0.977543	305	0.014605	214	0.962938
EPI4158283 / EPI2883780	Avian (Seagull) / Goose	H5N1 / H5N6	09-01	16.9444	3	0.996947	303	0.00209	182	0.994858
EPI4158283 / EPI2882776	Avian (Seagull) / Goose	H5N1 / H5N6	09-01	16.9444	3	0.996947	303	0.00209	182	0.994858
EPI4158283 / EPI4404191	Avian (Seagull) / Duck (Anas platyrhynchos)	H5N1 / H5N1	12-18	19.9385	3	0.996504	303	0.002655	127	0.993849
